# Translesion synthesis and microhomology-mediated end-joining repair in trypanosomatids

**DOI:** 10.1371/journal.pntd.0013626

**Published:** 2025-10-31

**Authors:** Lea Drogalis Beckham, Anne Snyder, Sylvie Doublié, Bruno Martorelli Di Genova

**Affiliations:** Department of Microbiology and Molecular Genetics, University of Vermont, Burlington, Vermont, United States of America; Ohio State University, UNITED STATES OF AMERICA

## Abstract

Trypanosomatid parasites and the diseases they cause affect more than 30 million people annually worldwide. To develop treatments for these diseases, it is critical to understand how trypanosomatid biology protects the parasite, so that these mechanisms may be exploited as drug targets. An important aspect of trypanosomatid survival is protection from oxidative damage inflicted by the host. Reactive oxygen species produced by the host can damage nuclear DNA and kinetoplast, the mitochondrion DNA. DNA damage must be repaired or bypassed for the trypanosomatid to continue to replicate its genome. Trypanosomatids also possess specialized redox pathways that neutralize reactive oxygen species (ROS) from host-derived attacks and endogenous mitochondrion metabolism. This Review Article focuses on how trypanosomatids employ microhomology-mediated end-joining to repair DNA double-strand breaks and translesion DNA synthesis to bypass oxidatively damaged bases in nuclear and kinetoplast DNA. While the deleterious effects of ROS must be managed to protect the parasite’s genome, the redox status generated by oxidative assault is crucial for intracellular signaling, DNA synthesis, and kinetoplast homeostasis. This Review will also comment on the effectiveness of current treatments for trypanosomatid-caused diseases and the role of oxidative damage in trypanosomatid diversity.

## Introduction

**Fig 1:**
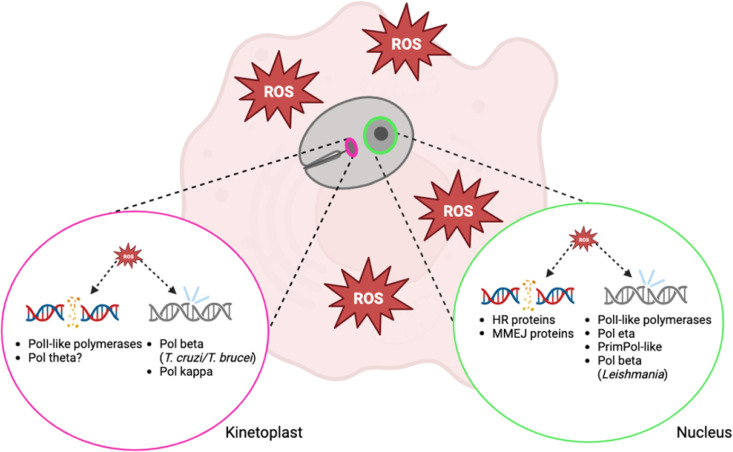
Trypanosomatid DNA double-strand break repair and translesion synthesis machinery in the kinetoplast (left) and nucleus (right). The kinetoplast relies on PolI-like DNA polymerases to repair double-strand breaks while the nucleus uses the full complement of homologous recombination and microhomology-mediated end-joining proteins. Oxidized bases are bypassed or repaired by different polymerases in the kinetoplast and the nucleus depending on the species of trypanosomatid.

Trypanosomatids are flagellated protozoan parasites of the class kinetoplastida. Known collectively as the Tritrypos [1], these parasites are the causative agents of neglected tropical diseases such as Chagas disease (*Trypanosoma cruzi*), human African trypanosomiasis (*Trypanosoma brucei*), and Leishmaniasis (*Leishmania* spp) [2,3]. Trypanosomatid-caused diseases affect over 30 million people worldwide annually and are a public health concern in Africa, Asia, and South America [4]. As protists, the trypanosomatids lack the advantage of coordinated multicellularity, thus their survival hinges on the ability of the individual parasite to maintain the integrity of its genome for future replication [5]. Oxidative damage is caused by the host immune response to infection. In this Review Article, we will discuss DNA repair pathways employed by trypanosomatids, which in some cases enhance proliferation and survival when the parasite is faced with oxidative attack (Fig 1).

## Repair of nucleobase damage

Oxidative stress is the direct consequence of living in an oxygen-rich environment. Reactive oxygen species (ROS) are the result of aerobic metabolism and are also generated by exposure to a number of external agents, including ionizing radiation or various drugs. For a recent review of ROS chemistry and its biological implications, please refer to [6]. ROS are a major source of damage to all macromolecules, including DNA. Nucleobase damage caused by oxidative stress or environmental factors may be repaired directly or bypassed during replication by specialized DNA polymerases that can incorporate nucleotides across DNA lesions in a process called translesion synthesis (TLS). Several repair mechanisms exist in Trypanosomatids to remove damaged bases. Nucleotide excision (NER) is a repair mechanism that removes bulky DNA damage induced by ultraviolet light, for example [7,8]. The mismatch repair pathway repairs mismatched base pairs that arise during replication [9,10]. The base excision repair (BER) pathway is responsible for repairing small, non-bulky lesions generated by oxidation, alkylation and deamination [11–13]. The remainder of this section will focus on repair of oxidized bases by BER enzymes. For a recent survey of Trypanosomatid MMR and NER proteins, please refer to [14].

During BER the damaged base is first recognized and excised by a DNA glycosylase, generating an abasic site or apurinic/apyrimidinic (AP) site. The DNA backbone is then cleaved by an AP lyase to create a single-strand nick. Next, apurinic endonuclease 1 (APE1) or polynucleotide kinase (PNK) processes the nick, leaving a free 3’ hydroxyl for Pol β to fill the gap. A DNA ligase then seals the newly added nucleotide [15–17].

7,8-dihydro-8-oxoguanine (8-oxoG) is a commonly described oxidized lesion derived from guanine [18,19]. It is repaired by OGG1 glycosylase in humans, AGOG in archaea, and Fpg and OGG2 in bacteria [20]. A putative 8-oxoguanine DNA glycosylase gene was found in the *T. cruzi* genome, TcOGG1 [21]. TcOgg1 was later found to be active in nuclear and mitochondrial BER [22]. *T. cruzi* also possesses a homolog of human MUTYH, a glycosylase specialized in detecting and excising dA in 8-oxoG:dA mispairs [23,24]. As for oxidized pyrimidine bases such as thymine glycol lesions, *T. cruzi* Nth1 does not appear to remove them, unlike its human counterpart, NTHL1 [25,26]. *T. cruzi* Nth1 acts instead as an AP endonuclease [27]. How the trypanosomatid repair Tg lesions and other common oxidized lesions such as Fapy-dG/dA [17,28] remains unknown.

As mentioned above, *T. cruzi* NTH1 does not repair Tg lesions, suggesting that another glycosylase or a TLS polymerase may be employed to resolve Tg lesions in trypanosomatids [25–27]. The Y-family [29] translesion synthesis (TLS) polymerases Rev1, Pol η (POLH gene) and Pol κ (POLK gene) are present in trypanosomatids, but Pol ι is apparently not represented. The only X-family polymerase is the base excision repair polymerase Pol β, as homologs of human Pol λ, μ, and TdT were not detected [15,30] (Table 1).

**Table 1:** Repair and translesion DNA Polymerases in *Leishmania major*, *Trypanosoma cruzi*, and *Trypanosoma brucei.*

Category	DNA Polymerase (Gene Name)	*Leishmania major* (Friedlin)	*Trypanosoma cruzi* (CL Brener)	*Trypanosoma brucei* (TREU927)
**Repair DNA Polymerases**	Pol β (POLB) (nuclear)	LmjF.08.0890 (homology; experimental in L. infantum [34])	TcCLB.503955.20, experimental [35,110]	Tb927.5.2780, experimental [36]
	Pol β-PAK (kDNA)	LmjF.08.0900 (homology)	TcCLB.503953.59, experimental [35,110]	Tb927.5.2790, experimental [36]
	Pol λ (POLL)	Absent	Absent	Absent
	Pol μ (POLM)	Absent	Absent	Absent
	TdT (DNTT)	Absent	Absent	Absent
	Pol θ (POLQ)	LmjF.24.0890 (homology; experimental in L. infantum [111])	TcCLB.508647.170, experimental [60]	Tb927.11.5550, experimental [40]
	Pol ν (POLN)	Absent	Absent	Absent
**Translesion DNA Polymerases**	Pol η (POLH)	LmjF.21.0630 (homology)	TcCLB.511911.120, experimental [42]	Tb927.10.1710 (homology)
	Pol ι (POLI)	Absent	Absent	Absent
	Pol κ (POLK)	LmjF.28.1410; LmjF.28.1420; LmjF.28.1430 (homology)	TcCLB.503755.30; TcCLB.503755.10, experimental [43]	Tb11.v5.0526; Tb11.v5.0661; Tb11.v5.0711; Tb11.v5.0756 (homology)
	REV1 (REV1)	LmjF.36.0100 (homology)	TcCLB.510963.10 (homology)	Tb927.10.4480 (homology)
	Pol ζ (REV3L)	LmjF.23.1330 (homology)	TcCLB.509769.130 (homology)	Tb927.8.3290 (homology)

In mammals, Pol β has been shown to localize to both the nucleus and mitochondria [31]. *Leishmania spp.* are unique in that they possess nuclear Pol β, which can perform base excision repair. No nuclear Pol β exists in *T. cruzi* and *T. brucei* [21,32], suggesting that other mechanisms are required to replicate nuclear DNA in the presence of oxidative lesions. In *T. cruzi* and *T. brucei*, Pol β is present exclusively in the kinetoplast [12,30,33–36]. An increase in endogenous Pol β expression in replicating *T. cruzi* epimastigotes in response to treatment with H_2_O_2_ has been reported, suggestive of BER activation in the kinetoplast [37]. Along with Pol β, *T. cruzi and T. brucei* possess Pol β-PAK, a Pol β-like enzyme in the kinetoplast that can participate in BER but is also able to perform TLS across from 8-oxoG [38]. No Pol β-PAK has been found in *Leishmania*, which is in keeping with the absence of mitochondrial Pol β in that species. The absence of all other X-family polymerases in trypanosomatids is of note due to their role in DNA repair. Without these repair enzymes, parasites may rely heavily on TLS polymerases to bypass damage. Furthermore, several X-family polymerases are involved in the processing of NHEJ substrates, which highlights their dispensability in an organism deficient in NHEJ [39].

*T. brucei* contains an A-family PolI-related translesion polymerase, PolIE, which localizes to the nucleus and functions in telomere maintenance [40]. A lack of nuclear Pol β in *T. cruzi and T. brucei* may drive reliance on translesion synthesis to overcome oxidative damage. Other error-prone polymerases like *T. brucei* PrimPol-like 2(PPL2) function late in DNA replication to do a final repair that allows completion of genome duplication [41].

Pol η is a Y-family translesion polymerase that can bypass 8-oxoG and has been extensively characterized in *T. cruzi* [30,42]. The POLH gene is duplicated in the *Leishmania* genus [30]. POLK gene amplication is seen in *Trypanosoma* and *Leishmania* spp. Pol κ has been shown to localize to the kinetoplast in *T. cruzi* epimastigotes where overexpression enhances parasite survival [43]. The maintenance of duplicated polymerase genes in trypanosomatids may be the result of pressure to repair DNA after oxidative assault by the host.

The trypanosomatid use of TLS to replicate damaged DNA may also enhance drug resistance. The trypanocidal drug Benznidazole induces DNA damage by oxidizing the nucleotide pool which is then incorporated into DNA. In *T. cruzi*, overexpression of repair polymerases including Pol β, κ, and η resulted in increased parasite resistance to Benznidazole treatment [44]. These findings indicate that lesion repair and bypass are mechanisms of drug resistance that can be targeted. In *T. brucei*, parasites depleted of TLS polymerase TbPolIE exhibited increased endogenous DNA damage and chromosomal segregation defects, demonstrating the critical role of TLS in parasite genomic maintenance [40]. Other potential drug targets include RNA polymerase TLS, the preferred mechanism for tolerating UV-induced lesions in *T. brucei* [7]. The importance of TLS in different contexts in trypanosomatids makes TLS polymerases compelling targets for drug development.

## Double strand break repair

Assaults on genomic integrity by reactive oxygen species result in damaged DNA nucleobases and DNA double-strand breaks (DSBs) [45]. There are several ways that ROS can lead to DSB formation. ROS-induced oxidized bases are generally repaired by the BER pathway. Excision of the damaged base by a DNA glycosylase is followed by the creation of a SSB. Two single-strand breaks (SSBs) in close proximity can generate a double-strand break (DSB). An oxidized base that is not repaired can block a replication fork, which can lead to replication fork collapse, and eventually produce a DSB if left unrepaired. DSBs are also produced in response to high levels of ROS when cells are exposed to ionizing radiation (IR). IR damages both DNA bases and sugar moieties, generating oxidized bases and ssDNA breaks. If two single-strand DNA breaks happen to be on opposite DNA strands within a few bases, a DSB can be generated [46].

**Fig 2:**
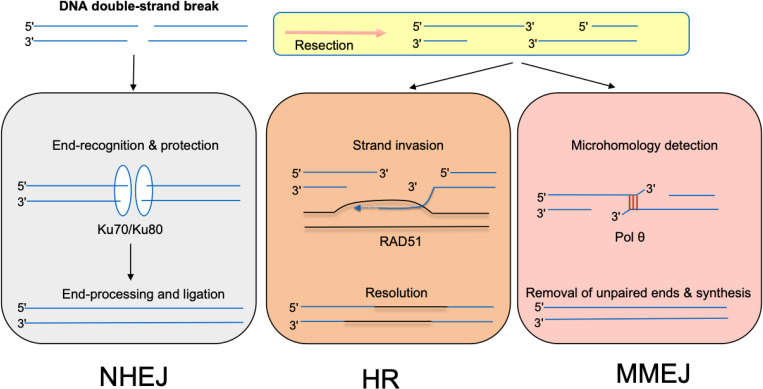
Double-strand break (DSB) repair pathways. *T. cruzi*, *T. brucei*, and *Leishmania* spp. lack the full complement of proteins necessary to carry out NHEJ, and thus rely on HR and MMEJ for DSB repair. NHEJ, non-homologous end joining; HR, homologous recombination; MMEJ, microhomology-mediated end joining. Figure adapted from [53].

DNA DSBs are particularly deleterious and must be repaired via one of several distinct pathways: non-homologous end joining (NHEJ), homologous recombination (HR), and microhomology mediated end-joining (MMEJ) [45] (Fig 2). In organisms that use NHEJ as a repair pathway, NHEJ is used predominantly to repair DNA DSBs during most of the cell cycle [46]. This repair process involves five core components: Ku70/Ku80 heterodimer (Ku), the DNA-dependent protein kinase catalytic subunit (DNA-PKcs), DNA ligase IV (Lig4), and the XRCC4 and XLF proteins. NHEJ is initiated by the binding of Ku70/80 heterodimers to DNA ends at a double-strand break. Ku then recruits DNA-PKcs. Ku70/Ku80 forms a stable complex with DNA-PKcs (DNA-PK), which promotes synapsis of the DNA ends. The final step of NHEJ is mediated by the LIG4 complex (DNA-Ligase 4, Xrcc4, and XLF). *Trypanosoma cruzi, T. brucei, and Leishmania spp*. do not possess the full complement of the proteins required to perform NHEJ: they possess Ku70/Ku80, but not Lig4. In these organisms DSBs are predominantly repaired via HR and MMEJ. The Ku70/80 complex might still participate in DSB repair as a “first responder” because of its ability to rapidly bind and protect DSBs. Nenarokova and colleagues hypothesize that the loss of NHEJ components may be responsible for characteristic features of parasite genomes [47].

DSBs that form during the S or G2 phase of the cell cycle are likely to be repaired predominantly by HR [48,49]. The HR repair process involves recombination between sister chromatids. It begins with the resection of the DSB 5’ DNA ends. This reaction is initiated by a trimeric complex, MRN, composed MRE11, RAD50, and Xrs2/NBS1. The resection event creates 3′-OH single-stranded DNA (ssDNA) tails that are covered by the single-stranded DNA binding protein RPA. RPA presents an obstacle to the loading of the RAD51 recombinase and has to be displaced by recombination mediator proteins like BRCA2 in order for HR to proceed. BRCA1 facilitates the recruitment of BRCA2 to DSBs through the bridging protein PALB2. The RAD51 filament promotes the search for a homologous template and enters the sister chromatid to form a D‐loop. The free 3′ end of the invading strand is then bound by a DNA polymerase, usually DNA polymerase δ (Pol δ), although translesion DNA polymerases have also been implicated in this process. Several of the core eukaryotic HR proteins are conserved among Trypanosomatids. Many parasites utilize HR to adapt to adverse environmental conditions and evade their host immune system via genetic recombination. For example, *T. brucei* uses HR to switch the expression of different Variant Surface Glycoproteins (VSG) genes and change its surface coat proteins [50].

Additionally, homologous recombination (HR) serves as a pivotal mechanism in *Leishmania*, facilitating genome maintenance, adaptability, and potentially antigenic variation. Central to this process is the RAD51 recombinase. Experimental disruption of RAD51-related genes in *Leishmania major* results in impaired DNA synthesis, defective replication, and heightened genomic instability, underscoring HR’s essential function in parasite survival [51]. Recent findings also highlight R-loops as significant contributors to genomic stability, revealing that their chromosome-size-dependent distribution profoundly influences DNA replication timing in *Leishmania* [52]. Loss of RNase H1, an enzyme crucial for resolving these RNA-DNA hybrids, further underscores their importance by disrupting replication timing and significantly enhancing chromosome instability and copy number variations [52]. These mechanisms, collectively involving HR, R-loop regulation, and recombination-based gene amplification, empower *Leishmania* to adapt to environmental stresses, including drug pressure, potentially facilitating immune evasion through antigenic variation, as evidenced by variations in surface antigens such as hydrophilic acylated surface proteins (HASPs) [53]. Collectively, these insights underline the multifaceted nature of HR and R-loop dynamics in governing genomic integrity and adaptability in *Leishmania*.

## Microhomology-mediated end-joining

The third DSB repair mechanism, microhomology-mediated end-joining (MMEJ), also happens after resection. It involves aligning microhomologies internal to broken DNA ends, trimming the DNA 3’-ends, and filling the remaining DNA gap by DNA polymerase θ. MMEJ is a mutagenic process that can result in insertions, deletions, and chromosomal rearrangements [54–57].

While HR is a mostly error-free DSB repair pathway, MMEJ is known to introduce genetic variability. A signature of double-strand break repair by MMEJ is that a few nucleotides are usually deleted or added at the repair site; this is the cost of using MMEJ to prevent extensive deletions and thus protecting the genome [56]. Pol θ, the enzyme at the heart of MMEJ, uses short DNA sequence homologies to initiate repair of double-strand breaks. Pol θ is found in all eukaryotes, except fungi [58]. In most eukaryotes Pol θ is composed of three domains, an N-terminal helicase-like domain and a C-terminal Family A DNA polymerase connected by a long central domain predicted to be mostly flexible [58]. The helicase-like domain grabs and aligns single-stranded DNA tails near the 3′ termini of the double strand break. The DNA polymerase domain then anneals the 3’-ends and proceeds with nucleotide addition. While the function of the central domain is still under active investigation, there is evidence that through its interactions with the globular domains, it plays in part in targeting the polymerase to specific locations and controlling the oligomeric state of the enzyme during repair [59]. Knowing the important roles that the central domain plays in other eukaryotes, it is intriguing that in Trypanosomatids the helicase and polymerase are not connected and are encoded by two separate genes [40,60]. Another notable difference is that the *T. cruzi* Polθ-helicase possesses both ATPase and helicase activity unlike its mammalian counterparts, which retain ATPase activity yet are not classical helicases because they do not unwind DNA under most conditions [40,60,61]. In mammals, Pol θ’s fusion of the helicase-like and polymerase domains could help facilitate a coordinated repair of double strand breaks. In contrast, the separation of the two domains in parasites may favor genome plasticity over fidelity. How the two separate protein gene products function together in Trypanosomatids during MMEJ remains to be elucidated [61,62].

MMEJ activity has been characterized in *T. brucei* [63] where it functions as an alternative to the preferred HR pathway [64,65]. Glover *et al.* determined that all intra-chromosomal joining observed after I-SceI treatment was due to MMEJ [65]. In *T. cruzi*, DSBs induced by CRISPR-Cas9 treatment were repaired exclusively via MMEJ when no HR template was available [66,67]. Similar breaks were found to be partially repaired via MMEJ in *L. donovani* [68]. In *L. donovani* cells with a disrupted RAD51 gene (required for HR), cells were reliant on MMEJ to repair CRISPR-Cas9 induced DSBs [69].

In *T. brucei*, antigenic variation is achieved through variant surface glycoprotein (VSG) expression, a mechanism that is critical for escaping the host immune system [70]. VSG genes are sub-telomeric, and the ability to change the singly expressed VSG hinges on the presence of DNA DSBs in the expression locus. Telomeric DSBs can trigger gene conversion through homologous recombination to switch between alternative VSG genes. In addition to repair by HR, MMEJ repairs ~25% of I-SceI induced DSBs in sub-telomeric VSG expression sites [64]. These regions often lack allelic homologous sequences with which to perform HR and are more reliant on functional MMEJ. This is in contrast to I-SceI induced breaks in the interior of the chromosome where only 10% of breaks were repaired by MMEJ [65].

DNA double-strand breaks that occur in mitochondrial DNA in other eukaryotic organisms can be repaired using MMEJ [71,72], and it might also be the case for trypanosomatids. Eukaryotic mitochondria lack NHEJ capability and repair using HR cannot account for the observed mutation rate in mitochondrial DNA. Therefore, trypanosomatids must also employ MMEJ to repair DSBs in kDNA. Four A-family polymerases that localize to the kinetoplast of *T. brucei* have been identified, termed PolIA-D [73–75]. Sequence analyses indicate that PolIB-D share a phage origin, whereas PolIA was derived from a Pol θ homolog [76]. While some work suggests that Pol θ may localize to mitochondria after H_2_O_2_ damage in human cell lines, more investigation is required to confirm this observation [77].

Outside of antigenic variation, repair of DNA DSBs is critical for maintaining genomic stability. The majority of DSBs in *Leishmania* are repaired via HR [68], but in the absence of MRN resection complex members MRE11 and RAD50, HR is diminished and chromosomal translocations were observed [53]. These translocations contained short micro-homologies at their junctions, indicating that the translocations were facilitated by MMEJ. Alternatively, in *T. brucei*, HR is dependent on the presence of RAD50 and the repair pathway preference switches to MMEJ in RAD50 null mutants [78]. The absence of RAD50 and MRE11 in *T. brucei* mutants did not result in a resection defect, suggesting that other nucleases may prepare DSBs for MMEJ. Using the trypanosomatid orthologue of single-strand binding protein RPA as a marker, Glover *et al.* showed that *T. brucei* can tolerate a high level of DNA damage during replication [79]. Taken together, these papers underscore how trypanosomatids balance HR and MMEJ carefully to maintain genomic integrity and promote adaptation to the host environment.

## Management of oxidative stress

The group *Kinetoplastida* is defined by the presence of a kinetoplast—a region within the cell’s only mitochondrion where its DNA is concentrated [80]. kDNA maxicircles encode the respiratory machinery and ribosomal RNA, typical of a mitochondrial genome. Minicircles encode guide RNAs that direct maxicircle transcript processing, making kDNA minicircle maintenance critical for mitochondrial function [81]. Mitochondrial respiration can generate oxidative stress in the kinetoplast, resulting in DNA damage, such as oxidized bases and double-strand breaks. Beyond the kinetoplast, nuclear trypanosomatid DNA is subjected to a similar oxidative assault by the host immune system [82]. Trypanosomatids also experience oxidative stress in the insect vector from both the insect’s immune system and parasite oxidation of vector-derived amino acids for energy [83]. The oxidative environments experienced by trypanosomatids throughout their life cycle makes the parasites vulnerable to DNA damage like oxidized bases and double-strand breaks (Fig 1).

Studies examining *T. brucei* DNA damage response to various genotoxic agents revealed that kDNA oxidative damage induced by H_2_O_2_ appeared at later experimental time points, indicating that the damage was caused indirectly. Regardless of the timing, *T. brucei* parasites from the human bloodstream repair kDNA damage better than parasites of the procyclic form from the Tsetse fly gut [84]. Faster kDNA repair in bloodstream *T. brucei* parasites demonstrates the mitochondrial demand from infectivity and superior fitness against oxidative attack. To prevent DNA damage in the face of oxidative stress—both host-inflicted and endogenously generated-- trypanosomatids possess a finely tuned redox management system that mitigates oxidative damage [85]. Despite this system, oxidative DNA damage still occurs. Possessing a single mitochondrion and enduring an oxidative onslaught requires trypanosomatids to prioritize repair mechanisms that maintain a complete genome. Repair of DNA DSBs using MMEJ and the bypass of DNA damage by TLS polymerases are both mutagenic mechanisms by which the parasite may maintain genomic integrity.

### Oxidative species as growth and differentiation signals

Moderate ROS can act as growth and differentiation cues that enhance proliferation and infectivity, whereas excessive ROS are deleterious [86–92]. Trypanosomatids experience an oxidative onslaught after infecting a host, but this attack does more than damage the parasite’s DNA. Reactive oxygen species and their intermediates have been demonstrated to function as signaling molecules [86] and are responsible for potentiating trypanosomatid infectivity in host cells [87–89]. In *T. cruzi*, exposure to pro-oxidant heme in the insect vector’s gut triggers an increase in ROS levels, promoting epimastigote proliferation. [90,91]. Treatment of *T. cruzi* with low concentrations of hydrogen peroxide (H₂O₂) has been observed to stimulate parasite proliferation, whereas higher concentrations are deleterious, leading to parasite death [92]. In *Leishmania* spp., ROS regulate differentiation from insect-stage promastigotes to replicative amastigotes, thereby enhancing virulence [93]. Oxidative stress-induced DNA damage serves as a driving force for intrastrain diversity in trypanosomatids. Exposure of *T. cruzi* epimastigotes to hydrogen peroxide (H₂O₂) resulted in a two-fold increase in mutation frequency [71], while *Leishmania donovani* promastigotes exhibited oxidative damage-associated mutations following sandfly infection [72].

Additionally, controlled oxidative stress has been shown to enhance the infectivity of *T. cruzi* by facilitating its survival and replication within host cells [87,92]. These findings suggest that moderate oxidative stress plays a key role in promoting genetic diversity, proliferation, and infectivity in trypanosomatids. However, excessive oxidative stress can be detrimental, underscoring the importance of a finely regulated balance between oxidative damage and parasite adaptation. Moderate oxidative stress not only enhances genetic diversity but may also serve as a replication checkpoint, delaying cell cycle progression in response to DNA damage [37]. This regulatory mechanism ensures that cells repair oxidized lesions before committing to further rounds of replication.

### Detoxification centered on trypanothione

**Fig 3:**
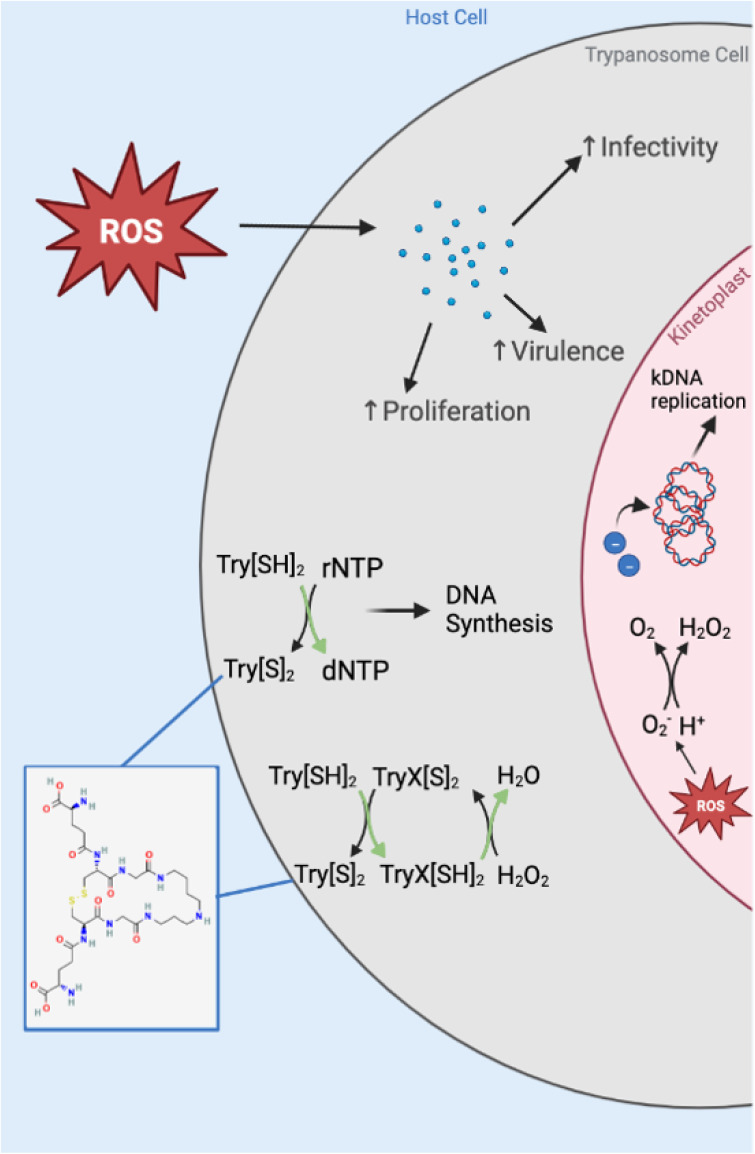
Oxidative stress management in trypanosomatids and role of trypanothione. TryS: trypanothione synthetase; TryX: tryparedoxin; rNTP: ribonucleoside triphosphate; dNTP: deoxynucleoside triphosphate; SOD: superoxide dismutase; APX: ascorbate peroxidase; RNR: ribonucleotide reductase.

The glutathione-derived thiol trypanothione (T[SH]₂/T[S]₂) organizes redox homeostasis and couples stress management to deoxynucleotide production via RNR, linking redox to DNA synthesis [94–97]. In humans, an increase in reactive oxygen species can be accompanied by a decrease in DNA synthesis to manage an excess of oxidative stress [94]. In this sense, redox status determines replication status. To manage oxidative stress, trypanosomatids possess an unusual form of the antioxidant glutathione composed of two glutathione molecules joined via a polyamine linker called trypanothione (Fig 3). In *T. brucei*, trypanothione supplies the reducing equivalents necessary for ribonucleotide reductase to convert ribonucleoside diphosphates into deoxyribonucleoside diphosphates, a critical step in DNA synthesis [95]. Since RNR is the rate-limiting enzyme in deoxynucleotide production, the trypanothione system is indispensable for maintaining DNA replication under oxidative stress [96]. The oxidative inactivation of RNR would impair DNA synthesis, suggesting that trypanosomatids must continuously regenerate their reducing environment to sustain proliferation. Further investigations in *T. brucei* revealed that genetic replacement of trypanothione-generating trypanothione synthetase (TryS) resulted in trypanosomatids that cannot establish an infection in mice and die when treated with TryS inhibitors [97]. Furthermore, overexpression of TryS results in increased growth and survival of *T. cruzi* epimastigotes and trypomastigotes, most notably in the presence of H_2_O_2_ induced oxidative stress [83]. *Leishmania infantum* has also been shown to rely on TryS activity to survive and replicate [98].

### Other detoxifying enzymes: SOD distribution and plant-like peroxidases

Beyond trypanothione metabolism, compartmentalized enzymes—superoxide dismutases (SODs) and peroxidases—buffer superoxide and peroxides across organelles [102–105]. The redox status of a trypanosomatid cell is also critical for kDNA replication. Maxicircles are replicated like other eukaryotic mitochondrial DNA, [99,100] but replicating the minicircles that edit them relies on redox balance. The universal minicircle sequence binding protein (UMSBP) must be reduced to activate its binding to kDNA minicircles and to initiate minicircle replication. Both the ability of UMSBP to bind DNA and oligomerize to become functional depend on the redox status of the cell; therefore, oxidative attacks by the host cell must be carefully balanced to protect the kinetoplast genome. Kinetoplast DNA (kDNA) is particularly susceptible to oxidative lesions due to its highly concatenated structure and constant exposure to reactive oxygen species [35]. The regulation of UMSBP activity through redox-dependent conformational changes ensures controlled initiation of minicircle replication, preventing excessive oxidative damage that could compromise mitochondrial function [101]. However, kDNA is also subjected to oxidative stress generated by the kinetoplast itself. Trypanosomatids possess several sites for potential electron leakage within their respiratory chain to form superoxide anions [102]. Several kinetoplast specific superoxide dismutases exist in trypanosomatids and inhibition of their activity in *T. cruzi* showed decreased parasitemia in mice [103], making superoxide dismutases an attractive target for treatment.

SOD isoforms are compartmentalized across the mitochondrion/kinetoplast, glycosomes, and cytosol in trypanosomatids, aligning with in vivo efficacy of iron-SOD inhibition [102].

For reactive oxygen species other than superoxide anions, trypanosomatids again rely on trypanothione oxidation. Tryparedoxin (TryX) is reduced by trypanothione and is in turn utilized by tryparedoxin peroxidase to decompose both endogenous- and macrophage-produced H2O2 and peroxynitrites as shown in *T. cruzi* [103], *T. brucei* [104], and *Leishmania* spp [105]. While trypanothione is synthesized in the cytosol, recent work has also revealed a trypanothione system in the kinetoplast that helps maintain mitochondrial redox homeostasis [106].

Most eukaryotes employ a catalase to decompose hydrogen peroxide to water and oxygen and to detoxify reactive oxygen species; however, no catalase homolog exists in human infective trypanosomatids [1,21,32,107]. Exogenous expression of *E. coli* catalase in *T. cruzi* trypomastigotes dysregulated the parasite’s response to oxidative stress, indicating that trypanosomatids have evolved a unique and highly tuned system (Fig 3) for managing reactive oxygen species [108,109]. In addition, trypanosomatids encode a plant-like ascorbate peroxidase (APX) activity rather than classical glutathione peroxidase/catalase dominance, reinforcing reliance on thiol-peroxidase chemistry [105].

Functionally, the absence of catalase appears adaptive: catalase would short‑circuit H₂O₂‑based signaling needed for proliferation/differentiation and would intersect poorly with the thiol‑peroxidase‑centric detox network. Expression of heterologous catalase dysregulates oxidative‑stress signalling and compromises development in trypanosomatids [1,21,32,107–109].

## Concluding remarks

DNA repair in eukaryotic cells necessitates specialized enzymes to maintain both nuclear and mitochondrial genomes. In trypanosomatids, this specialization is particularly pronounced because the kinetoplast serves as their sole mitochondrion. Due to their early divergence in evolutionary history, trypanosomatids have evolved extensive redundancy in DNA replication and repair mechanisms [104]. They possess a unique and comprehensive array of DNA replication and translesion synthesis (TLS) enzymes specifically adapted to ensure the integrity of kinetoplast DNA (kDNA) and nuclear DNA under persistent oxidative stress [105].

Parasite variability is also a capacity for environmental adaptation, not just a determinant of treatment outcome. HR‑ and MMEJ‑driven genome plasticity, R‑loop–regulated replication timing and copy‑number changes, and antigenic variation collectively enable transitions between mammalian hosts and insect vectors, tolerance of oxidative/nutritional stress, and immune evasion [52–54,69,78]. Therapeutic strategies that target conserved DNA repair–redox interfaces may therefore reduce both survival and evolvability across ecological niches.

While many front‑line agents (e.g., benznidazole, nifurtimox) exert trypanocidal activity by promoting oxidative or nitrosative stress, there are effective therapies and late‑stage candidates whose primary modes of action do not rely on direct free‑radical damage. Examples include inhibitors of polyamine/ornithine decarboxylase (e.g., eflornithine in HAT), disruption of sterol/ergosterol biosynthesis, membrane‑active amphotericin formulations and alkylphosphocholine drugs used in leishmaniasis (e.g., miltefosine), and emerging scaffolds that perturb proteostasis or organellar function; cytology‑based MoA profiling in trypanosomatids likewise reveals non‑oxidative mechanisms [4,111]. Recognizing these orthogonal routes clarifies that redox‑damage therapies are one pillar among several actionable vulnerabilities.

The protozoan parasites within the order Trypanosomatida display rapid evolutionary dynamics and considerable genetic divergence, both between species and among strains within the same species [106–108]. This genetic diversity poses significant challenges for treatment development, as slight variations in protein sequences across strains can affect drug efficacy. Effective therapeutic approaches must therefore account for this variability, targeting conserved mechanisms critical to parasite survival and replication.

Current trypanosomiasis treatments, including Benznidazole, Pentamidine, and Nifurtimox, primarily induce oxidative stress and DNA damage within parasites [109]. However, these drugs often fail to directly inhibit the parasite-specific DNA repair pathways, allowing parasites to mitigate damage, survive, and continue replication. Furthermore, many available treatments exhibit substantial off-target effects due to their broad-spectrum activity, resulting in undesirable side effects in hosts.

Identifying and exploiting the molecular differences in DNA repair mechanisms between trypanosomatids and their human hosts presents a promising strategy for developing highly selective therapies. Targeting parasite-specific DNA repair enzymes or pathways could enhance the efficacy of existing treatments and minimize host toxicity.

In conclusion, the complex interplay between oxidative stress and specialized DNA repair mechanisms underscores the importance of continued research into trypanosomatid biology. Elucidating the unique DNA maintenance strategies of these parasites will provide valuable insights into their evolution and adaptability, ultimately guiding the development of precise and effective therapeutic interventions that address the considerable genetic variability across trypanosomatid species and strains.

**Key learning points**

- Reactive oxygen species can damage DNA bases and, if left unrepaired, this damage can result in DNA double-strand breaks and lesions that lead to mutagenesis.
- Microhomology-mediated end-joining is an alternative DNA double-strand break repair pathway that facilitates antigenic variation and is critical for genome stability.
- Translesion synthesis allows trypanosomatids to complete DNA replication while tolerating mutagenic DNA damage.
- The redox status of the parasite cell is critical as it regulates processes like DNA replication and cell signaling through a glutathione-derived molecule exclusive to trypanosomatids, termed trypanothione.
- Current treatments for trypanosomatid-caused diseases target DNA integrity, but not the parasite’s ability to repair damage caused by the drug.
